# Therapeutic Effect of Buyang Huanwu Decoction on the Gut Microbiota and Hippocampal Metabolism in a Rat Model of Cerebral Ischemia

**DOI:** 10.3389/fcimb.2022.873096

**Published:** 2022-06-14

**Authors:** Rongmei Tang, Jian Yi, Shuangying Lu, Bowei Chen, Baiyan Liu

**Affiliations:** ^1^ The First Affiliated Hospital, Hunan University of Chinese Medicine, Changsha, China; ^2^ College of Integrated Traditional Chinese and Western Medicine, Hunan University of Chinese Medicine, Changsha, China

**Keywords:** cerebral ischemia, Buyang Huanwu decoction, gut microbiota, untargeted metabolomics, hippocampal metabolism, inflammation

## Abstract

Buyang Huanwu decoction (BHD) is a well-known Chinese herbal prescription. It has been widely used in the clinical treatment of cerebral ischemia (CI) in China. However, the mechanism underlying the treatment of CI with BHD remains to be elucidated. In this study, we combined microbiomic and metabolomic strategies to explore the therapeutic effects of BHD on middle cerebral artery occlusion (MCAO) in rats. Our results showed that BHD could effectively improve neurological severity scores and alleviate neuronal damage in rats with MCAO. BHD could also reduce the level of peripheral proinflammatory cytokines and inhibit neuroinflammation. 16S rRNA sequencing showed that BHD could increase the relative abundances of the genera *Lactobacillus*, *Faecalibacterium*, *Ruminococcaceae_UCG-002*, etc., while decreasing the relative abundances of the genera *Escherichia-Shigella, Klebsiella, Streptococcus, Coprococcus_2*, *Enterococcus*, etc. Untargeted metabolomic analysis of hippocampal samples showed that 17 significantly differentially abundant metabolites and 9 enriched metabolic pathways were linked with BHD treatment. We also found that the regulatory effects of BHD on metabolites were correlated with the differentially abundant microbial taxa. The predicted function of the gut microbiota and the metabolic pathway enrichment results showed that purine metabolism, glutamatergic synapses, arginine and proline metabolism, and alanine, aspartic acid and glutamate metabolism were involved in the effects of BHD. These pathways may be related to pathological processes such as excitotoxicity, neuroinflammation, and energy metabolism disorder in CI. In summary, these findings suggest that regulation of hippocampal metabolism and of the composition and function of the gut microbiota may be important mechanisms underlying the effect of BHD in the treatment of CI.

## Introduction

Stroke is a common and dangerous cerebrovascular disease. The incidence of cerebral ischemia (CI) accounts for 87% of all strokes, with high morbidity, a high disability rate and a high fatality rate, imposing many psychological and economic burdens on patients, families and society (Benjamin et al., 2018). CI injury can cause massive neuronal death. As adult neurons have a limited ability to proliferate or be replaced (Fricker et al., 2018), CI leads to severe neurological deficits in patients (Muir et al., 2007). At present, thrombolytic therapy is the main treatment for acute CI to salvage the ischemic penumbra, but its clinical application is greatly limited by a strict therapeutic time window (Prabhakaran et al., 2015). Therefore, the development of a safe and effective drug for the treatment of CI is a pressing challenge.

However, the injury and repair mechanisms of CI are very complex, involving a “waterfall-style” pathophysiological cascade caused by the interaction of multiple factors and mechanisms involving multiple targets and signaling pathways, causing massive neuronal loss and stress responses (Liao et al., 2017). Inflammation plays an important role in the pathological process of CI. The balance of inflammatory response is crucial for the brain injury and recovery (Jin et al., 2013). Inflammation could be a therapeutic target of CI (Jordán et al., 2008). Besides, some studies have also found metabolic alterations in CI and investigated the treatment for CI in the context of brain metabolism. Twenty metabolites, including lysine, glutathione have been reported as the biomarker for CI repeatedly. Some metabolic pathways, such as arginine biosynthesis; alanine, aspartate and glutamate metabolism may be involved in the development of CI (Jia et al., 2021). Modulation of metabolic changes in the cortex could provide neuroprotection (Liu et al., 2021).

Recently, researchers have found that there are bidirectional communications and interactions between the gut microbiota and the brain, known as the gut-brain axis. The gut-brain axis consists of vagus nerve, immune, and bacterial metabolites (Agirman and Hsiao, 2021; Agirman et al., 2021). CI could also be associated with the gut-brain axis. Patients with CI often experience gastrointestinal complications (Wen and Wong, 2017). Recently, with advances in human microbiota research, growing evidence indicates that CI is closely related to dysbiosis of the gut microbiota and that dysbiosis of the gut microbiota could exacerbate inflammatory responses (Yamashiro et al., 2017). For example, Benakis et al. observed that the changes in the gut microbiota induced by antibiotics can minimize the cerebral infarction area in mice with CI by increasing regulatory T cells (Tregs) and inhibiting γδT cells (Benakis et al., 2016). The bacterial metabolites could also contribute to the CI treatment. The bioactive metabolites from the gut microbiota such short-chain fatty acids and glutamate may participate the pathogenesis of CI (Chidambaram et al., 2022). SCFA-producing bacteria are associated with stroke outcome (Spychala et al., 2018). Therefore, the gut-brain axis may be a potential target for CI treatment.

Buyang Huanwu decoction (BHD) is a classic herbal formula of traditional Chinese medicine (TCM). BHD has been frequently applied in the clinic for the treatment of CI in China. The significant therapeutic effects of BHD in the acute phase, convalescence phase and chronic sequela phase of CI have been suggested by evidence-based medicine (Jiang et al., 2020). A previous study found that BHD can modulate the expression of VEGF and Flk1 and provide a therapeutic benefit for CI by promoting angiogenesis (Cai et al., 2007). Another study demonstrated that BHD could improve neurological function by promoting neurogenesis (Liu et al., 2013). The neurogenesis promotion by BHD is related to sirtuin 1/autophagy pathway (Li et al., 2021). However, as a TCM herbal formula, BHD contains many ingredients; its effects on CI could be the holistic result of the interactions among multiple components and multiple targets (Zhang et al., 2018). The therapeutic mechanism of BHD could be better illustrated by systemic methods. In a previous study, the neuroprotective mechanisms of BHD were explored by a genomic approach (Wang et al., 2011). A recent study combined proteomic and metabolomic methods to demonstrate the effects of BHD on neurodegenerative disease (Hsu et al., 2019). In addition, it has been reported that gut microbiota can metabolize TCM ingredients, and TCM can regulate the composition and homeostasis of the gut microbiota (Feng et al., 2019; Lin et al., 2021). However, knowledge of the link between the gut microbiota and metabolic phenotypes of CI and of the effects of BHD treatment for CI remains very limited.

In this study, we aim to explore the potential relationship between BHD treatment for CI and the gut microbiota. We proved the neuroprotection effect of BHD treatment. We employed 16S rRNA sequencing to study the gut microbiota changes in CI model after BHD treatment. We also used untargeted metabolomic analyses to investigate the metabolic mechanism underlying the effect of BHD in the treatment of CI. We found that BHD could modulate the composition of the gut microbiota. The function of the gut microbiota in rats with CI could also be modified by BHD treatment. BHD could also influence the metabolic profile of the hippocampus. The gut microbiota changes and metabolic pathway alterations could be correlated and have associations with inflammatory responses in CI. Our results demonstrate a novel mechanism underlying the effect of BHD in CI treatment.

## Materials and Methods

### Reagents

The nylon monofilaments (a2432A2) used for inducing middle cerebral artery occlusion (MCAO) stroke in a rat model were obtained from Beijing Cinontech Co., Ltd. Butylphthalide (NBP) soft capsules was obtained from CSPC Pharmaceutical Group Co., Ltd. Neuron-specific nuclear protein (NeuN) antibody (BM4354), ionized calcium binding adaptor molecule 1 (Iba-1) antibody (PB0517), and interleukin-1β (IL-11β), interleukin-6 (IL-6) and tumor necrosis factor alpha (TNF-α) enzyme-linked immunosorbent assay (ELISA) kits (EK0393, EK0412, EK0526) were purchased from Boster Biological Technology Co., Ltd. Sodium pentobarbital (P3761) was obtained from Merck KGaA (Darmstadt, Germany).

### Preparation and QC of BHD

BHD was prepared by mixing 120 g of *Astragalus mongholicus* Bunge, 6 g of *Angelica sinensis* (Oliv.) Diels, 5 g of *Paeonia lactiflora* Pall., 3 g of *Pheretima aspergillum* (E. Perrier), 3 g of *Ligusticum chuanxiong* Hort, 3 g of *Carthamus tinctorius* L. and 3 g of *Prunus persica* (L.) Batsch. All herbs constituting BHD were purchased from the First Affiliated Hospital of Hunan University of Chinese Medicine. These medicinal materials were immersed in five times the volume of distilled water for 1 h and then boiled for 2 h at 100°C. Then, the solution was filtered with 3 layers of medical gauze and concentrated to 2 g crude drug/mL.

For quality control (QC), the BHD extracts were analyzed using a UPLC–ESI–MS/MS system (UPLC, SHIMADZU Nextera X2, CBM30A system; MS, Applied Biosystems 4500 Q TRAP). The column was an Agilent SB-C18 column (1.8 µm, 2.1 mm*100 mm). Mobile phase A was water containing 0.1% formic acid, and mobile phase B was acetonitrile containing 0.1% formic acid. The elution gradient was as follows: 0 min, 5% B; 0-9 min, 5-95% B; 9-10 min, 95% B; 10-11.1 min, 95-5% B; 11.1-14 min, 5% B. The column temperature was 40°C, the flow velocity was 0.35 mL/min, and the injection volume was 4 µL. A triple quadrupole-linear ion trap mass spectrometer equipped with an electrospray ionization (ESI) Turbo Ion-Spray interface was used for LIT and triple quadrupole (QQQ) scans. The ESI source operation parameters were as follows: the source temperature was 550°C; the ion spray voltage was 5500 V (positive ion mode)/-4500 V (negative ion mode); and the ion source gas I, gas II, and curtain gas were set at 50, 60, and 25.0 psi. Instrument tuning and mass calibration were performed with 10 and 100 μmol/L polypropylene glycol solutions in QQQ and LIT modes. The QQQ scan was acquired *via* MRM experiments using medium nitrogen collision gas.

### Animals and Treatments

Male Sprague–Dawley rats (specific-pathogen-free grade; six weeks old; weight, 230-240 g) were purchased from Hunan Slike Jingda Experimental Animal Co., Ltd. (license number: SCXK 2019-0004). The rats were housed in groups of 4/cage. Housing conditions were maintained thermostatically at 22~26°C with 45~55% humidity and a 12−h light−dark cycle. The ventilation of the room remained constant throughout the day and night. Food and water were given freely.

The MCAO model was established according to a previously reported method (Longa et al., 1989). Rats were anesthetized with 0.3% sodium pentobarbital. The left common carotid artery (CCA), internal carotid artery (ICA) and external carotid artery were bluntly separated. The monofilament was fixed immediately when the black marker point on the monofilament was located at the CCA bifurcation, and then, the incision was sutured. Neurologic examinations were performed 2 hours after the surgery, with reference to Zea-Longa score reported by Longa, which indicated a successful model under the score from 1 to 3 (Longa et al., 1989).

NBP was used as a positive control drug for CI treatment (Wang et al., 2018). Based on our previous studies (Cai et al., 2007), we determined that the low dose of BHD (L-BHD) was 2.5 g/kg, the medium dose of BHD (M-BHD) was 5 g/kg, and the high dose of BHD (H-BHD) was 10 g/kg. Rats that received MCAO surgery were randomly divided into the model group, model+L-BHD (2.5 g/kg) group, model+M-BHD group (5 g/kg), model+H-BHD group (10 g/kg) and model+NBP (54 mg/kg) group. There were 14 rats in each group, and another 14 rats were used as the control group. After MCAO surgery, the rats in the BHD treatment groups and NBP group were intragastrically administered BHD or NBP once a day for 7 days, while the rats in the control group and the model group were intragastrically administered equal amounts of distilled water for 7 days. On the seventh day after the establishment of the MCAO models, the rats were subjected to a modified neurological severity score (mNSS) and then sacrificed. The experimental design is illustrated in **Figure 2A**. All of the above procedures were approved by the Ethics Committee of the Laboratory Animals of the First Affiliated Hospital of Hunan University of Chinese Medicine, and the ethics approval number was ZYFY20201215-1.

### mNSS Test

The neurological deficit scores of MCAO model rats were evaluated by the mNSS (Hu et al., 2017) with a score ranging from 0 to 18 points on the 7th day after ischemic injury. The mNSS mainly includes motor, sensory, balance, and reflex tests. Higher scores were correlated with more severe neurological function deficits.

### Sample Collection

All the rats were euthanized on the 7th day after MCAO surgery. Hippocampus isolated from rat brain tissue and intestinal contents (8 per group) were collected, frozen in liquid nitrogen for 30 min, and stored at -80°C. Serum samples (6 per group) were subpacked and stored at -80°C. Rat whole-brain tissues (4 per group) were fixed for histological staining.

### HE Staining

The whole brain tissue of the rats was excised, fixed in 4% paraformaldehyde, paraffin embedded and sectioned. After staining with hematoxylin and eosin (HE), the sections were examined under a light microscope (Leica, Germany) at a magnification of 20×, and images were captured.

### Immunohistochemical Staining

Immunohistochemical staining was performed on paraffin sections of rat whole brains to quantify NeuN- and Iba-1-positive cells on the ischemic side. After the sections were dewaxed at room temperature, they were treated with EDTA for heat-induced antigen retrieval, and then, 5% BSA was added, and the samples were incubated at 37°C for 30 min. The sections were incubated with primary antibodies against NeuN (1:150) and Iba-1 (1:100) overnight at 4°C. The sections were reheated at room temperature for 30 min, washed with PBS 3 times for 5 min each, and then incubated with the biotin-labeled goat anti-rabbit IgG secondary antibody for 30 min at room temperature. The sections were washed with PBS 3 times for 5 min each time, and SABC was added dropwise; then, the samples were incubated at 37°C for 30 min and then washed with PBS. DAB was used as the chromogenic substrate for color development, and then, sections were counterstained with Mayer’s hematoxylin. The sections were observed under a light microscope (Leica, Germany) at a magnification of 40×, and images were collected. The results were analyzed with ImageScope software and assessed using the histochemistry score (H-score) (Azim et al., 2015). The number of stained positive cells and their staining intensity (0, negative; 1, weak; 2, moderate; 3, strong) were recorded. H-score was calculated using the following formula: H score = (percentage of cells of weak intensity × 1) + (percentage of cells of moderate intensity × 2) + (percentage of cells of strong intensity × 3).

### Nissl Staining

The paraffin sections of rat whole brains were dewaxed to water routinely and then were incubated in 1% toluidine blue for 40min at 60°C. Subsequently, the sections were dehydrated in gradient ethanol, transparentized with xylene and sealed with neutral gum. The whole section was scanned using the Aperio VERSA 8 automated slide scanner (Leica, Germany), and the number of Nissl bodies in the cortical area were counted under 40× magnification in a blinded fashion.

### ELISA

ELISA was used to investigate the serum levels of IL-1β, CRP and TNF-α. All operations were completed in accordance with the corresponding ELISA kit instructions, and the absorbance was read at 450 nm.

### 16S rRNA Microbial Community Analysis

The MagPure Soil DNA LQ Kit (Magen, Guangdong, China) was used to isolate total genomic DNA from the intestinal content samples following the manufacturer’s instructions. After the DNA concentration and integrity were measured by a NanoDrop 2000 spectrophotometer and agarose gel electrophoresis, the V3-V4 hypervariable region of the bacterial 16S rRNA gene was amplified by PCR for amplicon sequencing library construction. Then, qualified libraries were sequenced on an Illumina NovaSeq6000 with two paired-end read cycles.

### Metabolomic Profiling Analysis

UPLC–MS/MS analysis was performed by OE BioTech (Shanghai, China). Hippocampal samples of rats were homogenized and centrifuged. The supernatant was kept for UPLC–MS/MS analysis. Hippocampal metabolic profiling was performed using a Dionex Ultimate 3000 RS UHPLC fitted with a Q-Exactive Plus Quadrupole-Orbitrap mass spectrometer equipped with a heated ESI source (Thermo Fisher Scientific, Waltham, MA, USA) in both positive and negative ion modes. Chromatography was performed on an ACQUITY UPLC HSS T3 column (1.8 μm, 2.1× 100 mm). The binary gradient elution system consisted of (A) water with 0.1% formic acid and (B) acetonitrile with 0.1% formic acid. Separation was achieved using the following gradient: 0-2 min, 5% B; 2-4 min, 5-30% B; 4-8 min, 30-50% B; 8-10 min, 50-80% B; 10-14 min, 80-100% B; 14-15 min, 100% B; 15-15.1 min, 100-5% B and 15.1-16 min, 5% B. The column temperature was 45°C. The flow velocity was 0.35 mL/min, and the injection volume was 2 µL. The mass range was from m/z 100 to 1,200. The mass spectrometer was operated as follows: spray voltage, 3,800 V (+) and 3,000 V (−); sheath gas flow rate, 35 arbitrary units; auxiliary gas flow rate, 8 arbitrary units; S-lens RF level, 50; Aux gas heater temperature, 350°C; capillary temperature, 320°C. QC samples were injected between every ten samples throughout the analytical run to evaluate the stability of the mass spectrometry system.

### Data Analysis and Statistics

For 16S rRNA sequencing, we removed the reads less than 50 bp in the original sequencing data then merged pairs of reads. The reads containing ambiguous bases or with homopolymer of > 8 bp were removed. The cleaned reads were clustered to generate operational taxonomic units (OTUs) with a 97% similarity threshold using VSEARCH (Rognes et al., 2016). Representative reads for each OTU were selected using the QIIME package (Caporaso et al., 2010) and annotated using the RDP classifier (Wang et al., 2007). Subsequently, microbial community structure was assessed by α (within-sample) diversity and β (between-sample) diversity analysis. PICRUST software was applied to predict microbial gene function based on the Greengenes data (Langille et al., 2013).

For untargeted metabolomic analyses, the original UPLC–MS/MS data were processed with Progenesis QI software (Nonlinear, Dynamics, Newcastle, UK) for baseline filtering, retention time correction, integration, peak alignment, peak identification and normalization. The metabolites were analyzed based on secondary fragments, accurate mass-to-charge ratio (m/z) and isotopic distribution using the HMDB database (Wishart et al., 2007). Principal component analysis (PCA) was utilized to determine the overall distribution of the hippocampal samples and the stability of the analysis process. Orthogonal partial least-squares-discriminant analysis (OPLS-DA) was used to screen differentially abundant metabolites. Metabolic pathway enrichment analysis was performed using the Kyoto Encyclopedia of Genes and Genomes (KEGG) database (https://www.kegg.jp/).

For statistical analysis, all data are expressed as the mean ± standard deviation (mean ± SD). The significant differences between groups were evaluated by one-way analysis of variance (ANOVA) and heteroscedasticity t-test using GraphPad Prism 8.0 software. Significant differences were accepted at p values <0.05.

## Result

### Identification of Chemical Constituents in BHD

**Figure 1:**
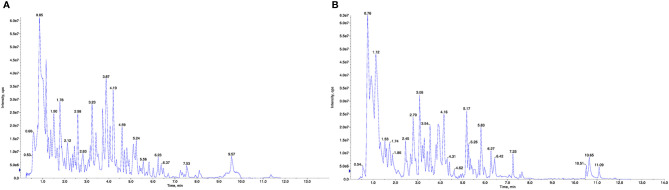
UPLC–ESI–MS/MS total ion chromatogram of BHD. **(A)** Positive ion mode. **(B)** Negative ion mode.

To identify the components in the BHD extracts, UPLC–ESI–MS/MS-based widely targeted metabolomics analysis was conducted for QC. The total ion chromatograms of a BHD sample are shown in **Figure 1**. In total, 1173 compounds were detected and identified, including 249 phenolic acids, 218 flavonoids, 61 terpenoids, 11 quinones, 46 lignans and coumarins, 2 tannins, 41 alkaloids, 115 organic acids, 61 lipids, 79 nucleotides and derivatives, 140 amino acids and derivatives, 1 steroid and 149 other compounds. Details of these compounds are shown in **Table S1**.

### BHD Improves Neurological Function and Ameliorates Neuronal Impairment in Rats With CI

**Figure 2:**
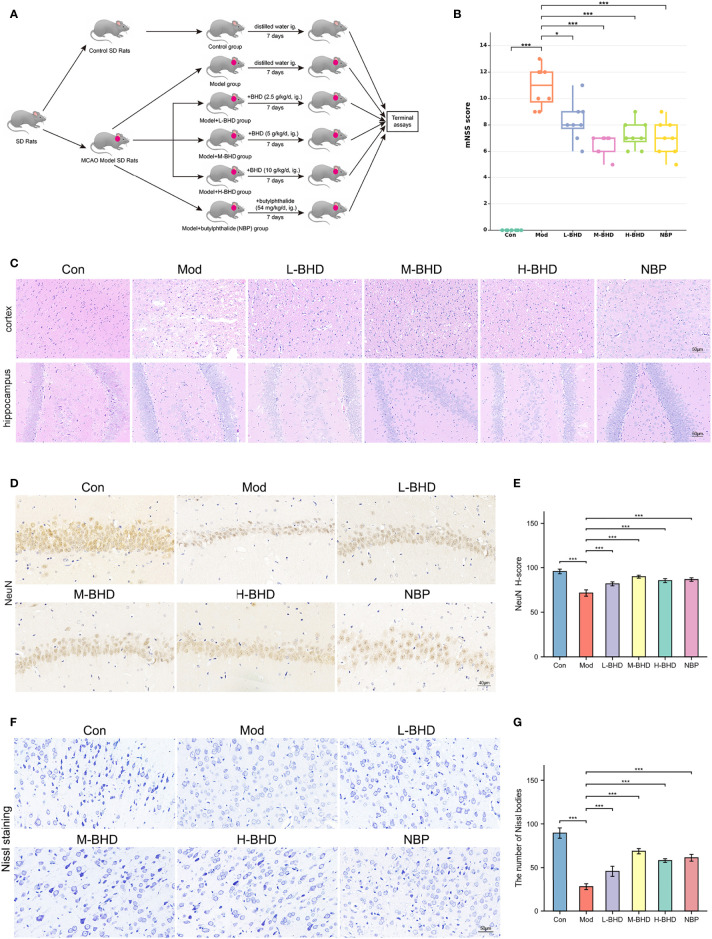
Effects of BHD on the modified neurological severity score (mNSS), pathological changes and neuronal damage of rats with CI. **(A)** Animal experimental design. **(B)** Comparison of mNSS between different groups (n = 8 per group). **(C)** HE staining at a magnification of 20× (n = 4 per group). **(D, E)** Immunostaining and quantification of NeuN-positive cells in the hippocampal CA1 region (n = 4 per group). **(F, G)** Nissl staining and cell counts of Nissl bodies in the cortical area (n = 4 per group). All the data are presented as the mean ± SD. Differences were assessed by one-way ANOVA and Student’s t-test: ***p < 0.001, *p < 0.05.

To evaluate the effects of BHD on the rats that received MCAO surgery, we employed the mNSS to rate the neurological functioning of rats and compared the mNSS among different animal groups. As shown in **Figure 2B**, the mNSS was increased significantly in the model group compared with the control group (*p*<0.001). Moreover, the mNSS was decreased significantly in the L-BHD, M-BHD, H-BHD and NBP groups compared with the model group (*p*<0.05), but there was no significant difference among the M-BHD, H-BHD and NBP groups. The results suggested that BHD could significantly promote the recovery of neurological function in rats with CI.

As shown by HE staining in **Figure 2C**, the number and volume of cells in the hippocampal dentate gyrus and cortex were reduced in the model group compared with the control group. The cells in the model group were also arranged irregularly and shrunken with a large number of surrounding vacuoles. However, compared with the model group, the injury in the groups treated with different doses of BHD and NBP was alleviated to varying degrees. The cell morphology in the treatment groups was also improved effectively, and the number of vacuoles was clearly reduced (**Figure 2C**).

Positive staining for NeuN (NeuN^+^) labels mature neurons. We next performed immunostaining to compare NeuN expression in the hippocampal CA1 region among different groups. As shown in **Figures 2D, E**, the density of NeuN^+^ cells in the hippocampal CA1 region in the model group was significantly lower than that in the control group (*p*<0.001), while the density of NeuN+ cells in the L-BHD, M-BHD, H-BHD and NBP groups increased significantly to varying degrees compared to that in the model group (*p*<0.001). There was no significant difference between the H-BHD and NBP groups, but the density of NeuN^+^ cells in M-BHD groups was significantly higher than the L-BHD, H-BHD and NBP groups(*p*<0.05).

Nissl bodies are large granular bodies found in neurons. Nissl staining was performed to stain Nissl bodies in survival neurons and evaluate the injury and metabolic ability of neurons in the cortical area. As shown in **Figures 2F, G**, Nissl bodies were large and abundant in the cortical area in the control group. Compared with the control group, Nissl bodies in the model group were sparsely arranged and significantly reduced in number and volume (*p*<0.001). Compared to the model group, the numbers of Nissl bodies were significantly increased to varying degrees in the L-BHD, M-BHD, H-BHD and NBP groups (*p*<0.001). There was no significant difference between the H-BHD and NBP groups, but the numbers of Nissl bodies in M-BHD groups were significantly higher than the L-BHD, H-BHD and NBP groups (*p*<0.05).

The above pharmacodynamic results indicated that BHD could effectively improve neurological function and ameliorate neuronal impairment in rats with CI. M-BHD (5 g/kg/d) had the best efficacy among the three BHD groups. Therefore, we used M-BHD as the representative treatment group to conduct subsequent gut microbiota and metabolomic studies.

### BHD Modulates the Overall Composition of the Gut Microbiota in Rats With CI

**Figure 3:**
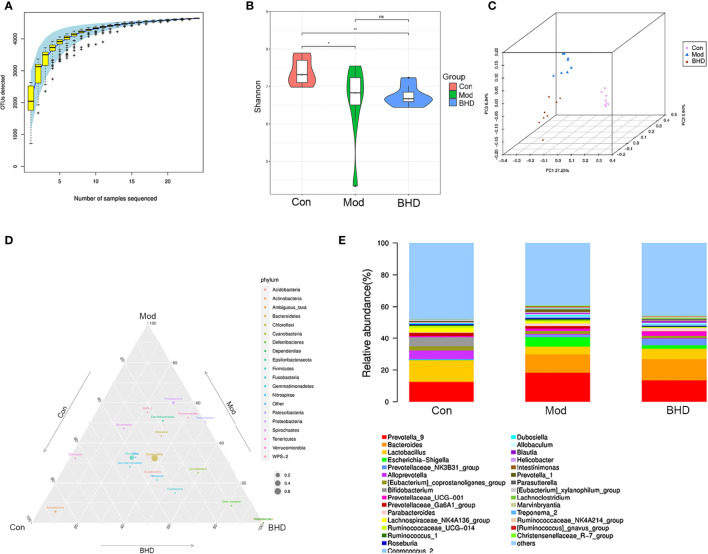
BHD modulated the overall structure and composition of the gut microbiota in rats with CI. **(A)** Species accumulation curve. **(B)** Shannon diversity index. **(C)** PCoA based on unweighted UniFrac distances. **(D)** Ternary phase diagram. **(E)** Community structure bar plot at the genus level. **p < 0.01, *p < 0.05. ns, not statistically significant. Con, control group; Mod, model group; BHD, BHD group. n = 8 per group.

To investigate the effects of BHD on the gut microbiota in rats with CI, we performed 16S rRNA sequencing analysis of the intestinal contents from the rats in the control, model and M-BHD groups. The high-quality sequences were clustered into OTUs at 97% similarity for species annotation analysis. The species accumulation curve (SAC) indicated that the amount of sequencing data was sufficient, and nearly 5000 kinds of gut microbes were detected. Thus, the sequencing analysis reflected the majority of microbial information in the three groups of samples (**Figure 3A**). The Shannon diversity index (SDI) was calculated. The SDI was changed in the rats with CI compared to the control rats, and BHD had no significant effect on gut microbiota α diversity in the rats with CI compared to the model group (**Figure 3B**). It indicates that the overall richness and diversity of species within the gut microbiota community (α diversity) are not changed by BHD compared to the model group. The β diversity was examined by principal coordinate analysis (PCoA) based on weighted UniFrac distances. There were significant differences in the microbiome composition among the three groups (ANOSIM analysis, *p*=0.001), which suggested that BHD could modulate the microbial composition of the gut microbiota in rats with CI (**Figure 3C**). As shown in the ternary phase diagram for the relative abundances of the top 20 phyla in the gut microbiota, *Bacteroidetes*, *Firmicutes* and *Proteobacteria* were the dominant phyla in all three groups, while Actinobacteria was more abundant in the control and BHD groups, and Deferribacteres and Dependentiae were more abundant in the BHD groups (**Figure 3D**). **Figure 3E** shows the community structure at the genus level. Here, the relative abundance of the top 30 genera in the gut microbiota of the three groups is shown. *Prevotella_9* and *Lactobacillus* were the dominant genera in the three groups. We observed that the proportion of *Prevotella_9* increased in the CI model groups, but the difference is not significant (*p*>0.05). *Lactobacillus* significantly decreased in the CI model group (*p*<0.05). In contrast, with BHD treatment, the proportion of *Prevotella_9* decreased, while that of *Lactobacillus* increased, compared with that in the model group, although the difference is not significant (*p*>0.05). The results suggested that microbial communities might play an important role in the effects of BHD treatment on rats with CI.

### Key Gut Microbiota Members Influenced by BHD in Rats With CI and Related Metabolic Function Prediction

**Figure 4:**
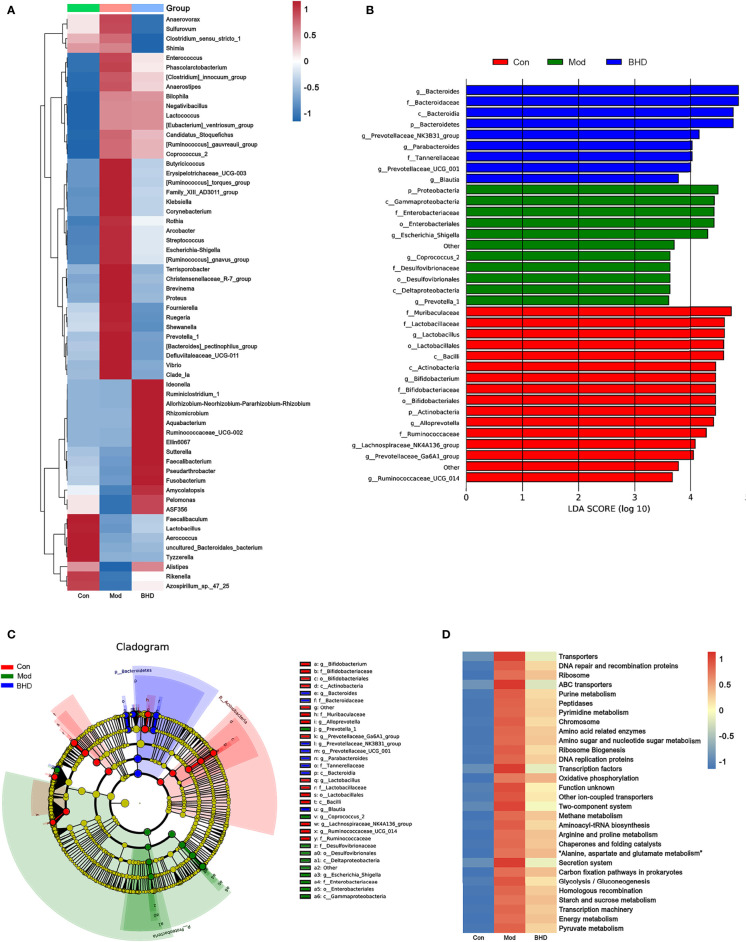
BHD affected the relative abundances of gut microbiota members in rats with CI. **(A)** Relative abundances of differentially abundant bacterial taxa across the three groups at the genus level. **(B)** LDA score distribution of differentially abundant bacterial taxa. **(C)** Cladogram of differentially abundant bacterial taxa. **(D)** Abundances of KEGG metabolic pathways of the gut microbiota. n = 8 per group.

To identify significantly differentially abundant microorganisms across the three groups, we used the Kruskal–Wallis test and found that 60 microorganisms showed significant changes at the genus level (*p*<0.05). Among the three groups, the relative abundances of the genera *Escherichia-Shigella, Klebsiella, Streptococcus, Coprococcus_2*, *Enterococcus*, etc., increased significantly after CI but decreased significantly after BHD treatment. The relative abundances of the genera *Lactobacillus*, *Faecalibacterium*, *Ruminococcaceae_UCG-002*, etc., decreased significantly after CI but increased significantly after BHD gavage (**Figure 4A**). At the family level, the relative abundances of Arcobacteraceae, Vibrionaceae and Enterobacteriaceae increased in the model group compared with the control group but decreased after BHD gavage (**Table S2**). In addition, we used the linear discriminant analysis (LDA) effect size (LEfSe) method (Segata et al., 2011) to identify the most differentially abundant taxa (LDA score>3 and *p*<0.05) among the three groups. The results indicated that there were 36 significantly differentially abundant microorganisms at the phylum (n=3), class (n=5), order (n=4), family (n=11) and genus (n=14) levels. In the BHD group, Bacteroides (from the phylum to genus levels) had the highest LDA scores. The LDA scores of Prevotellaceae_NK3B31_group, Parabacteroides and Prevotellaceae_UCG_001 were also high in the BHD group (**Figures 4B, C**).

Subsequently, PICRUSt was employed to further explore the effects of BHD on the metabolic function of the gut microbiota. As predicted from the 16S rRNA profile, 203 KEGG metabolic pathways were significantly affected in the CI model group compared with the control group (*p*<0.05), and their levels tended to be partially restored after BHD gavage (**Table S3**). In **Figure 4D**, the total abundances of the top 30 metabolic pathways (KEGG Level 3) are shown. Compared with the control group, the abundances of purine metabolism, glutamatergic synapse, arginine and proline metabolism, and alanine, aspartic acid and glutamate metabolism were upregulated significantly in the CI model group, and the abundances of these metabolic pathways decreased significantly after BHD gavage (**Figure 4D** and **Table S3**).

### Effects of BHD on the Microbiota May Be Related to Inflammatory Responses

**Figure 5:**
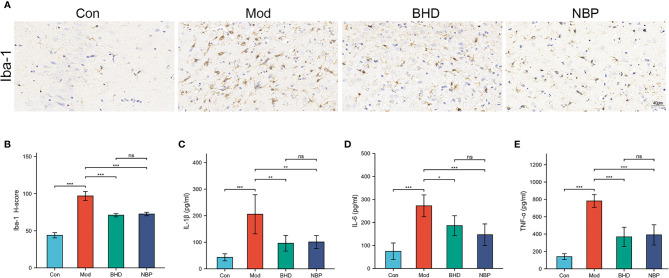
Effects of BHD on microglia in the brain and peripheral proinflammatory cytokines in rats with CI. **(A, B)** Quantification of Iba-1-positive cells in the ischemic brain tissue of rats (n = 4 per group). **(C-E)** Serum levels of IL-1β, IL-6, and TNF-α (n = 6 per group). All the data are presented as the mean ± SD. Differences were assessed by one-way ANOVA: ***p < 0.001, **p < 0.01, *p < 0.05. ns, not statistically significant. n = 8 per group.

The gut microbiota could affect multiple host organs including brain by modulating the host immune system (Benakis et al., 2016). Microglia play an important role in neuroinflammation (Kwon and Koh, 2020). To examine the effects of BHD on inflammatory responses, microglia in the brain tissue of the rats were labeled with the specific marker Iba-1. Compared with the control group, the number of Iba-1+ cells in the ischemic side brain tissue of the rats in the model group increased, and the cell body became larger (*p*<0.001), indicating that the microglia were transformed from the resting state to a highly activated state. However, intervention with BHD significantly inhibited microglial activation (*p*<0.001). Previously studies show that the gut microbiota could influence brain physiology and pathology by the abnormal production of inflammatory cytokines (Rutsch et al., 2020). We tested three peripheral proinflammatory cytokines, namely, IL-1β, TNF-α and IL-6 by ELISA. The levels of IL-1β, TNF-α and IL-6 in the serum of rats with CI increased significantly (*p*<0.001) compared with the control group, while BHD treatment significantly reduced their levels (*p*<0.05, **Figures 5C–E**). The above results implied that BHD could affect the gut microbiota and modulate the inflammatory responses of rats with CI.

### Identification of Brain Metabolites and Related Metabolic Pathways Influenced by BHD Treatment in Rats With CI

**Figure 6:**
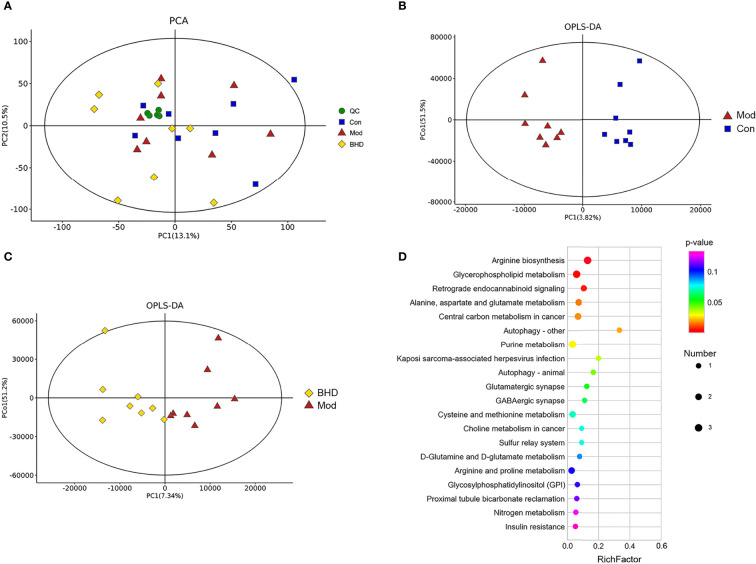
BHD regulated hippocampal metabolism in rats with CI. **(A)** Hippocampal metabolite analysis of the three groups based on PCA. QC, quality control. **(B)** Score plots of OPLS-DA between the control and model groups. **(C)** Score plots of OPLS-DA between the BHD and model groups. **(D)** Bubble diagram of the metabolic enrichment pathways based on the differentially abundant metabolites among the three groups. The x-axis shows the Rich factor, the color of the circle indicates the *p value*, and the size of the circle reflects the number of differentially abundant metabolites in each pathway. n = 8 per group.

Changes in the trajectories of brain metabolites are widely involved in a vast range of biological processes after CI. To characterize the brain metabolic changes caused by BHD, we determined the comprehensive metabolic profile of the brains of all the rats by UPLC–MS/MS. We focused on the hippocampus because it is an important area in the brains of adult mammals and is involved in neurogenesis, promoting neurorepair and affecting cognitive function (Lisman et al., 2017; Marques et al., 2019). PCA was used to reflect the overall differences in metabolic profiles among the three groups. As shown in **Figure 6A**, there was a strong trend of separation between the BHD group and the other two groups. The findings suggested that the structure of metabolites in the hippocampus of rats with CI was changed after BHD treatment. Next, OPLS-DA was used to identify biomarkers of different groups. As seen in **Figures 6B, C**, the metabolic characteristics of the control group, the model group and the BHD treatment group were significantly different. The hippocampal metabolites in different groups were identified based on a variable importance of projection (VIP) value >1 and a t test with *p*<0.05. According to this qualification, 34 metabolites showed significant changes among the three groups (**Table 1**). Among them, 17 metabolites showed significant changes between the BHD group and the model group. Another 17 metabolites showed significant changes between the control group and the model group. They also presented an opposite trend in the BHD group. Compared with the model group, the levels of D-fructose 2,6-bisphosphate, sedoheptulose 1,7-bisphosphate, alpha-D-glucose 1,6-bisphosphate, L-acetylcarnitine, L-glutamine, N2-succinyl-L-ornithine, adenine, D-glucurono-6,3-lactone, 1-methyladenosin and zidovudine were significantly reduced in the BHD group (*p*<0.05). BHD treatment also tended to reduce the levels of maltol, PC (15:0/16:1(9Z)), butyrylcarnitine, PE (16:1(9Z)/22:4(7Z,10Z,13Z,16Z)), D-glucose, O-acetylserine, N6,N6,N6-trimethyl-L-lysine, 5’-methylthioadenosine, creatine and pyroglutamic acid, which were significantly increased in the model group compared to the control group. On the other hand, glycolic acid, glyceraldehyde, argininosuccinic acid, asparaginyl-valine, 3’-AMP, trans-cinnamic acid and 3a,6b,7b-trihydroxy-5b-cholanoic acid were significantly enriched in the BHD group compared with the model group. BHD treatment also had a certain callback effect on the levels of N-methylethanolaminium phosphate, S-(formylmethyl)glutathione, methylisocitric acid, citrulline, succinyladenosine, phenethylamine glucuronide and N-acetylneuraminic acid, which were significantly decreased in the model group compared to the control group.

**Table 1:** The differential metabolites in the hippocampus after BHD treatment.

Formula	Metabolites	VIP		Trend	
		Mod/Con	BHD/Mod	Mod/Con	BHD/Mod
**C_6_H_14_O_12_P_2_**	D-Fructose 2,6-bisphosphate	4.3	3.6	↑*	↓*
**C_7_H_16_O_13_P_2_**	Sedoheptulose 1,7-bisphosphate	1.8	1.6	↑*	↓*
**C_6_H_14_O_12_P_2_**	Alpha-D-Glucose 1,6-bisphosphate	2.8	2.9	↑*	↓*
**C_9_H_17_NO_4_**	L-Acetylcarnitine	8.0	2.5	↑***	↓*
**C_5_H_10_N_2_O_3_**	L-Glutamine	3.1	2.1	↑***	↓*
**C_2_H_4_O_3_**	Glycolic acid	2.4	2.3	↓**	↑*
**C_3_H_6_O_3_**	Glyceraldehyde	2.5	1.1	↓*	↑*
**C_9_H_16_N_2_O_5_**	N2-Succinyl-L-ornithine	2.3	1.7	↑**	↓*
**C_4_H_9_N_3_O_2_**	Creatine	17.1	12.1	↑**	↓
**C_5_H_7_NO_3_**	Pyroglutamic acid	6.8	1.1	↑**	↓
**C_11_H_19_NO_9_**	N-Acetylneuraminic acid	4.9	1.6	↓**	↑
**C_11_H_15_N_5_O_3_S**	5’-Methylthioadenosine	2.5	1.5	↑*	↓
**C_14_H_19_NO_6_**	Phenethylamine glucuronide	1.7	0.6	↓*	↑
**C_9_H_20_N_2_O_2_**	N6,N6,N6-Trimethyl-L-lysine	1.8	0.8	↑***	↓
**C_7_H_10_O_7_**	Methylisocitric acid	1.9	0.3	↓***	↑
**C_14_H_17_N_5_O_8_**	Succinyladenosine	2.5	1.6	↓*	↑
**C_10_H_13_N_5_O_4_**	Zidovudine	0.7	2.4	↑	↓**
**C_43_H_76_NO_8_P**	PE (16:1(9Z)/22:4(7Z,10Z,13Z,16Z))	32.1	19.1	↑*	↓
**C_9_H_8_O_2_**	trans-Cinnamic acid	1.5	4.1	↓	↑*
**C_12_H_19_N_3_O_7_S**	S-(Formylmethyl)glutathione	1.3	0.3	↓*	↑
**C_11_H_15_N_5_O_4_**	1-Methyladenosine	0.8	1.2	↑	↓*
**C_5_H_9_NO_4_**	O-Acetylserine	3.6	3.6	↑*	↓
**C_6_H_12_O_6_**	D-Glucose	2.5	1.3	↑**	↓
**C_10_H_14_N_5_O_7_P**	3’-AMP	1.1	1.1	↓	↑*
**C_11_H_21_NO_4_**	Butyrylcarnitine	1.4	0.1	↑***	↓
**C_6_H_8_O_6_**	D-Glucurono-6,3-lactone	0.4	1.1	↑	↓**
**C_9_H_17_N_3_O_4_**	Asparaginyl-Valine	0.7	1.6	↓	↑*
**C_6_H_6_O_3_**	Maltol	2.0	1.5	↑*	↓
**C_39_H_76_NO_8_P**	PC (15:0/16:1(9Z))	20.1	12.5	↑*	↓
**C_5_H_5_N_5_**	Adenine	1.8	2.5	↑	↓**
**C_3_H_10_NO_4_P**	N-Methylethanolaminium phosphate	1.3	0.6	↓*	↑
**C_6_H_13_N_3_O_3_**	Citrulline	1.3	0.8	↓*	↑
**C_10_H_18_N_4_O_6_**	Argininosuccinic acid	0.8	1.2	↓	↑*
**C_24_H_40_O_5_**	3a,6b,7b-Trihydroxy-5b-cholanoic acid	1.1	0.7	↓**	↑*

↑, content increased; ↓, content decreased; ***p < 0.001, **p < 0.01, *p < 0.05.

To explore the functions of the differentially abundant metabolites, we analyzed the metabolic pathways in which the metabolites were involved based on the KEGG database. The top 20 metabolic pathways ranked according to significance are shown in **Figure 6D**. Nine metabolic pathways with *P*<0.05 were selected as potentially enriched pathways for BHD intervention in CI, including arginine biosynthesis, D-glutamine and D-glutamate metabolism, glycerophospholipid metabolism, retrograde endocannabinoid signaling, alanine, aspartate and glutamate metabolism, central carbon metabolism in cancer, autophagy – other, purine metabolism, Kaposi sarcoma-associated herpesvirus infection and autophagy – animal. Some of these enriched metabolic pathways were similar to the metabolic pathways of the gut microbiota predicted by PICRUSt analysis, such as purine metabolism, glutamatergic synapse, arginine and proline metabolism, and alanine, aspartic acid and glutamate metabolism. This indicated the possibility that the effects of BHD treatment on brain metabolites and the gut microbiota might be associated.

### Potential Relationships Between Hippocampal Metabolites and the Gut Microbiota

**Figure 7:**
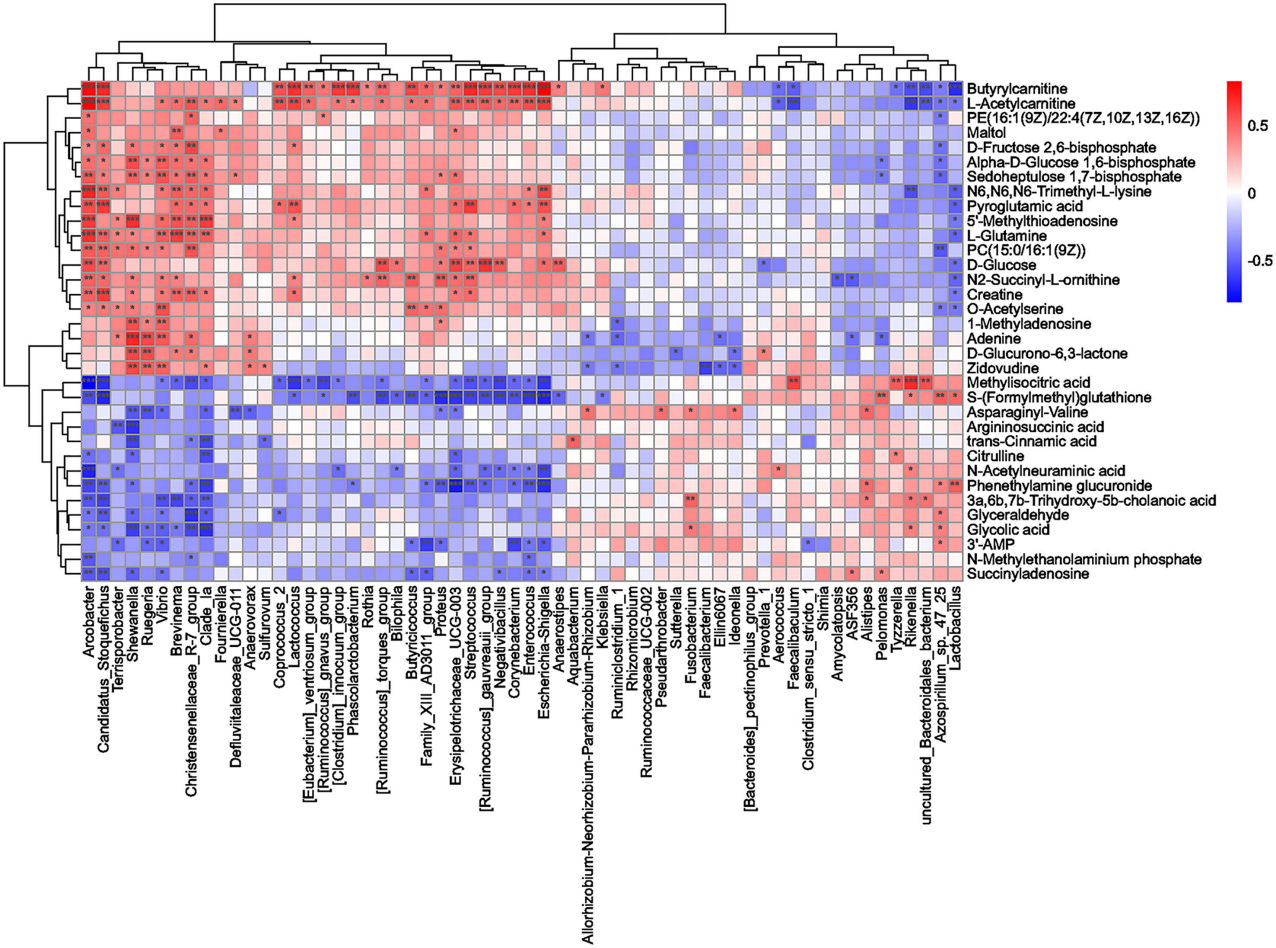
Correlation analysis of differentially abundant microbial taxa and differentially abundant hippocampal metabolites. Spearman correlation coefficients were used to generate the matrix. The degree of correlation is shown by the gradient change in colors; red denotes a positive correlation, and blue denotes a negative correlation. ***p < 0.001, **p < 0.01, *p < 0.05.

To further investigate the potential relationship between the changes in hippocampal metabolites and the gut microbiota in rats with CI after BHD treatment, the correlations among 34 differentially abundant metabolites and 60 differentially abundant microbial species were measured by Spearman correlation. The results are shown in **Figure 7**. *Shewanella*, *Vibrio*, *Brevinema*, *Arcobacter*, *Streptococcus*, *Lactobacillus* and *Escherichia-Shigella* were significantly correlated with multiple metabolites (*p*<0.05). This suggested that there is a potential complex relationship between the gut microbiota and brain metabolites. BHD may affect the communication between the gut and brain by modulating the gut microbiota as well as hippocampal metabolites in rats with CI, which may play an important role in the mechanism underlying the effect of BHD in the treatment of CI.

## Discussion

BHD contains diverse chemical components, and the different components can affect different targets in various pathways. However, most previous studies could not systematically and comprehensively clarify the characteristics of the efficacy of BHD. In this study, we investigated the therapeutic mechanism of BHD on CI using a rat MCAO model. First, our results suggest that BHD could effectively improve neurological function and alleviate neuronal damage in rats with MCAO, which confirmed that BHD had a therapeutic effect on CI. Second, we explored the therapeutic mechanism of BHD by an omics method from holistic and systemic perspectives, which is suitable for the multitarget and multilink nature of TCMs such as BHD. Specifically, the gut microbiota has been shown to be related to CI through its interaction with the host immune system (Benakis et al., 2016). This study revealed a novel connection between the therapeutic mechanism of BHD and the gut microbiota by 16S rRNA sequencing. The data suggest that BHD not only modulates the overall structure and function of the gut microbiota in rats with CI but also affects inflammatory responses. Furthermore, through metabolomic profiling of the hippocampus, we revealed that BHD could influence the metabolic pathways in rats with CI. We also found a potential relationship between hippocampal metabolites and the gut microbiota in rats with CI after BHD treatment. Therefore, our study demonstrates a novel mechanism for the therapeutic effects of BHD on CI.

In the present study, 16S rRNA sequencing results showed that the relative abundance of Enterobacteriaceae increased significantly in the gut of rats with CI, while its abundance significantly decreased after BHD treatment. A previous study showed that Enterobacteriaceae is an independent risk factor for the early recovery of CI patients, and inhibiting the growth of Enterobacteriaceae can reduce systemic inflammation, ameliorate hippocampal neuron injury and alleviate CI in MCAO model mice (Xu et al., 2021). At the genus level, 60 microbial taxa with significant differences were found; some of these taxa may affect the therapeutic effects of BHD. In particular, the relative abundances of the genera *Escherichia-Shigella*, *Klebsiella*, *Streptococcus*, *Coprococcus_2* and *Enterococcus* increased significantly in the gut of rats with CI, while BHD gavage significantly decreased their relative abundances. Among them, *Escherichia-Shigella*, a typical member of *Enterobacteriaceae* with high pathogenicity and transmissibility, can increase intestinal permeability, produce a large amount of endotoxin, and induce a systemic inflammatory response (Soares et al., 2012; Small et al., 2013; Belotserkovsky and Sansonetti, 2018). Studies have found that *Escherichia-Shigella* can induce the production of proinflammatory cytokines by activating the NLRP3 inflammasome (De la Fuente et al., 2014; Liu et al., 2020), and its abundance is positively correlated with the levels of the proinflammatory molecules CXCL2, IL-1β, and NLRP3 (Cattaneo et al., 2017). *Klebsiella* species are among the main pathogenic bacteria that causes pneumonia, sepsis, meningitis and other diseases (Pitout et al., 2015; Mashni et al., 2019). A recent study demonstrated that overgrowth of *Klebsiella* in the gut was significantly associated with severe brain damage in premature neonates through proinflammatory polarization of immune cells and dysregulation of the gut-microbiota-immune-brain axis (Seki et al., 2021). *Streptococcus* is a large genus of Gram-positive bacteria that can cause severe inflammatory responses or autoimmune diseases (Syed et al., 2020). A study found that *Coprococcus_2* is a characteristic genus in the feces of patients with obesity and polycystic ovary syndrome (Zhou et al., 2020). *Enterococcus* is a genus of important pathogens that cause nosocomial infection, which can lead to infectious diseases such as meningitis, septicemia and abdominal infection (Raza et al., 2018). On the other hand, BHD could increase the relative abundance of *Faecalibacterium. Faecalibacterium* can produce butyric acid, affect the differentiation of colonic regulatory T cells, inhibit histone deacetylation to reduce the levels of the proinflammatory cytokines IL-1β, TNF-α and IL-6, and attenuate NF-κ B-mediated NLRP3 signaling to exert anti-inflammatory effects (Glauben et al., 2006; Furusawa et al., 2013; Parada Venegas et al., 2019). It has also been found that supplementation with butyric acid in the gut can alleviate neuroinflammation and neurotoxicity and play a beneficial role in the treatment of Parkinson’s disease (Sharma et al., 2015). In sepsis, the disappearance of *Faecalibacterium* and other symbiotic bacteria is accompanied by an increase in the relative abundances of drug-resistant pathogens such as *Enterococcus* in the gut microbiota (Ravi et al., 2019). This dysbiosis is similar to our results. We found that compared with the control group, the relative abundance of *Enterococcus* increased significantly and that of *Faecalibacterium* decreased significantly in the gut microbiota of rats with CI, whereas BHD gavage reversed these changes. In addition, BHD gavage also significantly increased the relative abundances of the genera *Lactobacillus* and *Ruminococcaceae_UCG-002*, which significantly decreased in the model group. *Lactobacillus* is a kind of intestinal probiotic that has been proven to help repair damaged intestinal barrier function (Liu et al., 2011) and plays an important role in regulating human immune function and maintaining human health (Hirano et al., 2003; Vlasova et al., 2013). In addition, a study reported that an increase in *Lactobacillus* abundance can help inhibit neuronal apoptosis, decrease cerebral infarction volume and reduce oxidative stress in rats with CI (Wanchao et al., 2018). It has been reported that the abundance of *Ruminococcaceae_UCG-002* is significantly reduced in the gut of patients with hyperglycemia during pregnancy (Gao et al., 2020) or in patients with ulcerative colitis (Liu et al., 2021). Therefore, the low level of *Ruminococcaceae_UCG-002* may have an impact on host gut dysbiosis.

The above analyses show that disorder of the gut microbiota after CI can lead to an increase in the abundance of pathogenic bacteria and a decrease in the abundance of beneficial bacteria. Dysbiosis may be an important factor triggering a systemic inflammatory response and exacerbating neuroinflammation in the brain. Microglia, the innate immune cells in the brain, are rapidly activated after stroke and gather at the site of cerebral infarction to release inflammatory mediators, which are an important part of the neuroinflammatory response after stroke (del Zoppo et al., 2007; Du et al., 2021). Microglia can receive signals from the gastrointestinal tract under disease conditions, and gut microbiota intervention can affect the maturation and function of microglia and participate in the regulation of neuroinflammation (Erny et al., 2015; Abdel-Haq et al., 2019).In this work, we found that the number of Iba-1-positive cells increased significantly in ischemic brain side tissue, and their cell bodies became larger. This suggested that microglia were rapidly activated and accumulated at the infarct site, exacerbating neuroinflammation. Our results showed that this change could be reversed by BHD treatment. We also found that the levels of serum IL-1β, TNF-α and IL-6 were elevated significantly. Previous studies have found that the levels of these proinflammatory cytokines increase after CI, contributing to the systemic inflammatory response and exacerbating cerebral ischemic injury (Ferrarese et al., 1999; Lambertsen et al., 2012). After BHD treatment, the abundances of intestinal pathogens decreased, while those of beneficial bacteria increased, and the levels of the proinflammatory cytokines IL-1β, TNF-α and IL-6 in serum were significantly downregulated. Based on the above results, we hypothesize that BHD could modulate the disordered gut microbiota after CI and alleviate the inflammatory response. Thus, the gut microbiota might be a new therapeutic target for BHD in the treatment of CI. However, the specific mechanisms involved in the regulation of inflammatory responses by the gut microbiota after CI remain to be further explored.

Metabolomics can be used to systematically reveal the mechanisms of diseases as well as the possible therapeutic mechanisms of drugs (Schrimpe-Rutledge et al., 2016). The complex host–microbial co-metabolism is also closely related to various diseases (Heinken and Thiele, 2015). Many host metabolites are affected by the interactions of the gut microbiota and host cells (Lai et al., 2021). In this study, we used UPLC–MS/MS analysis to explore the metabolites in the hippocampus of rats and identified 17 differentially abundant metabolites. We found some common pathways, such as purine metabolism, glutamatergic synapse, arginine and proline metabolism, and alanine, aspartic acid, and glutamate metabolism, in both the predicted functions of the gut microbiota and the KEGG metabolic pathways of the hippocampal metabolome. Through correlation analysis, it was found that some differentially abundant microbial taxa and some differentially abundant metabolites were highly correlated. Next, we will discuss some potential relationships between hippocampal metabolites and the gut microbiota in detail.

Glutamatergic synapse and glutamate metabolism were both enriched for the hippocampal metabolites and preidcted from 16S rRNA profile. Glutamate is one of the most important excitatory neurotransmitters. Excitotoxicity is the primary factor that causes damage to neurons after CI. Ischemia and hypoxia can stimulate massive accumulation of extracellular glutamate and trigger a cascade of delayed neuronal degeneration and death (Krzyżanowska et al., 2014; Lai et al., 2014). Glutamine is a precursor of glutamate. Studies have shown that maintaining the optimal concentration and ratio of glutamine and glutamate in the brain during CI is essential for the survival of neurons, astrocytes and normal nervous system function (Stelmashook et al., 2011). In this study, we observed a significant increase in the L-glutamine level in the hippocampus of rats with CI, suggesting that the glutamate-glutamine cycle was enhanced. BHD significantly decreased the L-glutamine content, which may alleviate the excitotoxic injury of neurons by indirectly inhibiting the production and release of glutamate. Furthermore, we found that the abundance of *Lactobacillus* was negatively correlated with the level of L-glutamine (correlation coefficient=-0.48, *p*<0.05). This observation implies the modulation of the L-glutamine level by *Lactobacillus* may contribute to the effect of BHD treatment.

In addition, we found that the concentration of pyroglutamic acid increased significantly after CI compared to that in the control group and decreased after BHD treatment. The metabolic pathway involved in pyroglutamate is the glutathione metabolism which significantly affected in the CI model group (**Table S3**). Pyroglutamic acid can be cleaved by 5-oxoprolinase to produce glutamate and regulate the concentration of glutamate in the γ-glutamyl cycle (Niehaus et al., 2017). Excessive accumulation of pyroglutamate can lead to high anion gap metabolic acidosis (Serpa et al., 2018) and can cause protein oxidation and the production of active substances in the brains of rats to damage the antioxidant defense of the brain and promote oxidative stress (Pederzolli et al., 2007; Pederzolli et al., 2010). In addition, we found that the level of pyroglutamic acid was positively correlated with *Candidatus_Stoquefichus* (correlation coefficient=0.64, *p*<0.001) and negatively correlated with *Lactobacillus* (correlation coefficient=-0.49, *p*<0.05). *Candidatus_Stoquefichus* was significantly increased in the model group (*p*<0.01). It needs further study for the role of these genera in the metabolism of pyroglutamic acid and their relationships with the effect of BHD treatment.

The microbiota related to purine metabolism pathway significantly increased in the model group and decreased in BHD group (*p*<0.05). Energy metabolism disorder caused by CI is an important factor leading to neuronal injury (Sveinsson et al., 2014). Purine plays multiple roles in brain injury. During metabolic stress induced by CI, adenosine is released and mobilized (Dale and Frenguelli, 2009) to inhibit energy expenditure, neurotransmitter release and neuronal firing, increase nutrient supply (Borea et al., 2018) and promote the recovery of transmembrane sodium ion gradients (Attwell and Laughlin, 2001), which is beneficial to the energy budget of metabolically stressed neurons. In the purine metabolic pathway, 3’-AMP, as the precursor of adenosine, can be converted to adenosine (Verrier et al., 2012). Adenosine can be cleaved to form adenine (Cader et al., 2020). *Family_XIII_AD3011_group* was significantly correlated with the level of 3’-AMP (correlation coefficient=-0.61, *p*<0.01). We found that BHD significantly decreased the abundance of *Family_XIII_AD3011_group* and increased the level of 3’-AMP (*p*<0.05). BHD also significantly reduced the level of adenine and the abundance of *Shewanella* that were upregulated in the hippocampus of rats with CI. The level of adenine and the abundance of *Shewanella* were correlated (correlation coefficient=0.70, *p*<0.001). Thus, BHD may regulate the level of adenosine and improve energy metabolism disorders in CI by modulate the abandunce of *Family_XIII_AD3011_group* and *Shewanella.*

Arginine metabolism was both related to the hippocampal metabolites and the gut microbiota. The inflammatory response is a typical pathological feature of CI. Intestinal malabsorption disorders caused by intestinal failure and decreased intestinal blood flow under inflammatory responses such as endotoxemia may be involved in the decrease in citrulline and arginine levels (Luiking et al., 2009; Kao et al., 2013). Citrulline and aspartate are condensed under the catalysis of argininosuccinate synthetase to produce argininosuccinic acid, the immediate precursor of arginine (Rochovansky and Ratner, 1967). Argininosuccinic acid is catalyzed by argininosuccinate lyase to form arginine (Ratner et al., 1953). It has also been found that oral administration of L-citrulline can reduce the death of hippocampal neurons in mice after transient cerebral ischemia (Yabuki et al., 2013; Matsuo et al., 2017). In this study, the concentration of citrulline decreased significantly in the model group. Argininosuccinic acid was significantly upregulated in the BHD group. It is suggested that the occurrence of disorder of the arginine biosynthesis pathway is related to CI. BHD treatment may alleviate the inflammatory response by regulating the arginine biosynthesis pathway. In addition, BHD treatment significantly decreased the abundance of *Shewanella* which was negatively correlated with the level of argininosuccinic acid (correlation coefficient=-0.65, *p*<0.001). The influence of BHD on *Shewanella* may also be a way to promote the absorption of arginine.

## Conclusion

In summary, BHD has therapeutic effects on MCAO in rats by effectively improving neurological function, alleviating neuronal damage, reducing the level of peripheral proinflammatory cytokines, and inhibiting neuroinflammation. Our study found that BHD could modulate the overall structure and function of the gut microbiota and lead to a decrease in pathogenic bacteria and an increase in beneficial bacteria in rats with CI. BHD could also affect hippocampal metabolism pathways, such as purine metabolism, glutamatergic synapse, and arginine biosynthesis. There may be potential links between changes in gut microflora and changes in hippocampal metabolism. This study provides new ideas and a preliminary experimental basis for the pharmacodynamic effects of BHD after CI.

## Data Availability Statement

The datasets presented in this study are publicly available in online repositories. The names of the repository/repositories and accession number(s) can be found below: NCBI [accession: PRJNA808830]; Metabolights [accession: MTBLS4366].

## Ethics Statement

The animal study was reviewed and approved by the Ethics Committee of the Laboratory Animals of the First Affiliated Hospital of Hunan University of Chinese Medicine, and the ethics approval number was ZYFY20201215-1.

## Author Contributions

BL and RT conceived the original idea and designed the experiment. RT, SL, and JY finished the experiments. RT, JY, and BC performed the statistical analysis. RT wrote the manuscript and BL reviewed the manuscript. All authors contributed to the article and approved the submitted version.

## Funding

This work was supported by grants from the National Natural Science Foundation of China (82074251).

## Conflict of Interest

The authors declare that the research was conducted in the absence of any commercial or financial relationships that could be construed as a potential conflict of interest.

## Publisher’s Note

All claims expressed in this article are solely those of the authors and do not necessarily represent those of their affiliated organizations, or those of the publisher, the editors and the reviewers. Any product that may be evaluated in this article, or claim that may be made by its manufacturer, is not guaranteed or endorsed by the publisher.
